# *In vivo* effects of the pure aryl hydrocarbon receptor antagonist GNF-351 after oral administration are limited to the gastrointestinal tract

**DOI:** 10.1111/bph.12576

**Published:** 2014-03-18

**Authors:** Zhong-Ze Fang, Kristopher W Krausz, Kenjiro Nagaoka, Naoki Tanaka, Krishne Gowda, Shantu G Amin, Gary H Perdew, Frank J Gonzalez

**Affiliations:** 1 Laboratory of Metabolism, Center for Cancer Research, National Cancer Institute, National Institutes of HealthBethesda, MD, USA; 2 Department of Pharmacology, Pennsylvania State University College of MedicineHershey, PA, USA; 3 Center for Molecular Toxicology and Carcinogenesis, Department of Veterinary and Biochemical Sciences, Pennsylvania State UniversityUniversity Park, PA, USA

**Keywords:** GNF-351, metabolic map, absorption, AHR antagonist

## Abstract

**Background and Purpose:**

GNF-351 is a potent aryl hydrocarbon receptor (AHR) antagonist that inhibits dioxin response element-dependent and independent activities. Here, the absorption, metabolism and *in vivo* AHR antagonist activity of GNF-351 were investigated.

**Experimental Approach:**

LC-MS metabolomics was used to analyse GNF-351 metabolism *in vitro* and *in vivo*. Recombinant drug-metabolizing enzymes were employed to determine the enzymes involved in GNF-351 metabolism. Analysis of target AHR genes was performed to investigate the inhibitory effects of GNF-351 towards AHR activation.

**Key Results:**

Several phase I metabolites were generated after GNF-351 was incubated with microsomes from human or mouse liver and intestine, including two oxidized GNF-351 and one tri-demethylated GNF-351. Poor absorption from the intestine resulted in no detectable levels of GNF-351 in mouse serum (0–6 h) and urine (24 h) and almost all GNF-351 was found in the faeces after 24 h. Analysis of faeces further revealed all the *in vitro* phase I metabolites. Novel metabolites were detected, including one di-oxidized GNF-351, two oxidized and tri-demethylated GNF-351, one dehydrogenated product of oxidized GNF-351, and one sulfation product of di-oxidized GNF-351. Cytochromes P450 were demonstrated to be the major enzymes involved in metabolism of GNF-351. After oral administration to mice, GNF-351 readily inhibited β-naphthoflavone-induced AHR activation in ileum and colon, but not that in the liver.

**Conclusion and Implications:**

While poor absorption and extensive metabolism after oral administration limited the *in vivo* effects of the pure AHR antagonist GNF-351 in liver, it could be used to inhibit AHR activation in intestine and colon.

## Introduction

**Figure 1:**
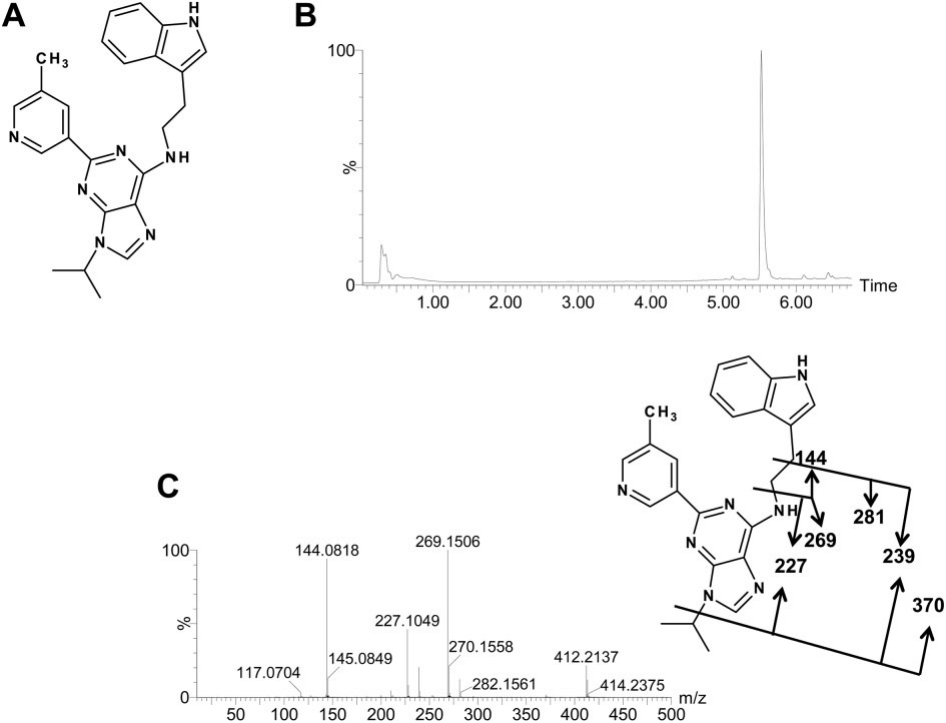
Chromatography and MS/MS fragmentation of GNF-351. A. The structure of GNF-351; B. Chromatographic separation of GNF-351; C. MS/MS fragmentation pattern of GNF-351.

Aryl hydrocarbon receptor (AHR), a basic-helix-loop-helix Per-Arnt-Sim transcription factor, has a key role in xenobiotic-induced toxicity and carcinogenesis (Barouki *et al*., 2007; Vondracek *et al*., 2011; receptor nomenclature follows Alexander *et al*., 2013). The most high-affinity AHR agonist is the notorious environmental and industrial toxin 2,3,7,8-tetrachlorodibenzo-*p*-dioxin (TCDD) and TCDD toxicity is due to AHR activation (Fernandez-Salguero *et al*., 1995; Matsubara *et al*., 2012). Development of AHR antagonists has become an important topic in recent years because of their potential therapeutic role. For example, the AHR antagonist 2-methyl-2H-pyrazole-3-carboxylic acid prevented TCDD-associated toxicity (Kim *et al*., 2006). Additionally, AHR antagonists can attenuate Th17 cell development *in vivo* and subsequent secretion of IL-17 and IL-22 (Veldhoen *et al*., 2009). GNF-351 (Figure 1A) is a recently developed AHR antagonist with the capacity to inhibit dioxin response element-dependent and-independent activity. Additionally and in contrast to other reported antagonists, such as α-naphthoflavone, GNF-351 does not exhibit partial agonist potential (Smith *et al*., 2011).

Pharmacokinetic properties are important determinants for the research and development (R&D) of the therapeutic agents, including the absorption and elimination characteristics, and poor pharmacokinetics will limit the development of drugs for clinical applications. For example, the clinical use of rifaximin is limited to gastrointestinal diseases because of its poor absorption (Ma *et al*., 2007). In contrast, the closely related antibiotic rifampicin has excellent bioavailability and is used to treat systemic infections such as tuberculosis (Li *et al*., 2013)

Ultra performance liquid chromatography-electrospray ionization-quadrupole time-of-flight mass spectrometry (UPLC**®**-ESI-QTOFMS)-based metabolomics has become an important tool to determine the metabolism, toxicity and metabolic behaviour of xenobiotics (Johnson and Gonzalez, 2012; Johnson *et al*., 2012). The present study used metabolomics to elucidate the absorption behaviour and the main metabolic pathway of GNF-351, including identification of metabolites and the major drug-metabolizing enzymes (DMEs) involved in metabolism. The influence of absorption and metabolism of GNF-351 on use of GNF-351 as an efficient AHR antagonist *in vivo*, was also examined.

## Methods

### Animals

All animal care and experimental procedures complied with protocols approved by the National Cancer Institute Animal Care and Use Committee. The mice were maintained under a standard 12 h light, 12 h dark cycle with water and chow provided *ad libitum*. All studies involving animals are reported in accordance with the ARRIVE guidelines for reporting experiments involving animals (Kilkenny *et al*., 2010; McGrath *et al*., 2012). A total of 22 animals were used in the experiments described here.

### *In vitro* metabolism of GNF-351 in liver microsomes (LMs), intestine microsomes (IMs) and recombinant DMEs

Livers from untreated 6-to 8-week-old male C57BL/6J mice were homogenized to prepare microsomes (MLM) as previously described (Fang *et al*., 2012). Human liver microsomes (HLM) were purchased from BD Gentest Corp. (Woburn, MA, USA). Human intestinal microsomes (HIM) were purchased from BD Gentest Corp. (Woburn, MA, USA). Mouse intestinal microsomes (MIM) were prepared as described for mouse liver microsomes. The phase I incubation system (200 μL) contains 50 mM Tris-HCl buffer solution (pH = 7.4), 0.5 mg mL^−1^ HLM, MLM, HIM or MIM, 5 mM MgCl_2_, 100 μM GNF-351, and 1 mM freshly prepared NADPH. After 0.5 h incubation at 37°C, the reaction was stopped using 200 μL cold 50% aqueous methanol containing 5 μM chlorpropamide. After centrifuging at 14 000× *g* for 20 min, a 5 μL aliquot of the supernatant was injected into a UPLC-ESI-QTOFMS.

The *in vitro* incubation system for recombinant phase I enzymes was similar to the microsomal incubation system. Recombinant cytochrome P450 (CYP) 1A1, CYP1A2, CYP2C8, CYP2C9, CYP2D6, CYP2A6, CYP2B6, CYP3A5, CYP3A4, CYP2C19, CYP2E1, flavin monooxygenase (FMO)-1, FMO-3, FMO-5, UDP-glucuronosyltransferase (UGT) 1A3 and UGT1A4 produced in baculovirus, were purchased from BD Gentest Corp. CYPs, 2 pmol, and 5 μg FMOs were incubated with 100 μM of GNF-351. The reaction time was 30 min and metabolites were analysed using UPLC-ESI-QTOFMS.

For investigation of GNF-351 glucuronidation, the incubation system (200 μL) contained 50 mM Tris-HCl buffer solution (pH = 7.4), 0.5 mg mL^−1^ HLM or MLM, 25 μg mL^−1^ alamethicin, 5 mM MgCl_2_, 100 μM GNF-351, 1 mM D-saccharic 1,4-lactone, and 1 mM freshly prepared uridine 5′-diphosphoglucuronic acid (UDPGA). The same incubation system was used for screening the UGT isoforms involved in the glucuronidation of GNF-351. The concentration of UGT isoforms used was 0.1 mg mL^−1^, and the incubation time was 30 min.

### *In vivo* treatment of mice with GNF-351 and sample preparation

Eight 6-to 8-week-old male C57BL/6J mice supplied by The Jackson Laboratory (Bar Harbor, ME, USA) (four control and four GNF-351-treated mice) were used to investigate the metabolism of GNF-351 *in vivo*. GNF-351, dissolved in corn oil, was administered by oral gavage at a dose of 5 mg·kg^−1^ body weight. Control mice were treated with the same volume of corn oil alone. The serum (0–6 h), urine (24 h) and faeces (24 h) were collected for analysis. Urine and faeces samples were collected using metabolic cages (Metabowls, Jencons Scientific USA, Bridgeville, PA, USA), and blood samples collected in BD microtainer serum separator tubes (Franklin Lakes, NJ, USA) by retroorbital bleeding. Serum samples were centrifuged for 15 min at 8000× *g*, and 10 μL serum was diluted with 190 μL 66% aqueous acetonitrile containing 5 μM chlorpropamide. Urine samples prepared by mixing 20 μL urine with 180 μL 50% aqueous acetonitrile containing 5 μM chlorpropamide. The faeces were pulverized and 1:20 50% aqueous acetonitrile (5 μM chlorpropamide) added for extraction followed by centrifugation. All samples were centrifuged at 14 000× *g* for 20 min, and 5 μL aliquot of the supernatants was injected into a Waters UPLC-ESI-QTOFMS system (Waters Corporation, Milford, MA, USA).

### UPLC-ESI-QTOFMS

An Acquity C18 BEH UPLC column (Waters Corporation) was employed to separate components in serum, urine, faeces and microsomal incubation samples. The mobile phase consisted of water containing 0.1% formic acid (A) and acetonitrile containing 0.1% formic acid (B). The following gradient condition was used: 100% A for 0.5 min, increased to 100% B over the next 7.5 min and returned to 100% A in the last 2 min. The flow rate of mobile phase was set 0.5 mL min^−1^. Data were collected in positive ion mode on a Waters Q-Tof Premier mass spectrometer, which was operated in full-scan mode at 50–850 m/z. Nitrogen was used as both cone gas (50 L·h^−1^) and desolvation gas (600 L·h^−1^). Source desolvation temperatures were set at 120 and 350°C respectively. The capillary voltage and cone voltage were 3000 and 20 V respectively. The structures of metabolites were elucidated by tandem MS fragmentography with collision energies ranging from 15 to 40 eV.

### Multivariate data analysis

MarkerLynx software (Waters Corporation) was used to deconvolute the chromatographic and mass spectrometric data. A multivariate data matrix containing information on sample identity, ion identity (retention time and m/z), and ion abundance was generated through centroiding, deisotoping, filtering, peak recognition and integration. The data matrix was further analysed using SIMCA-P^+^ 12.0 software (Umetrics, Kinnelon, NJ, USA). Orthogonal partial least squares data analysis (OPLS-DA) was adopted to analyse the data to identify the major latent variables in the data matrix. Potential metabolites were identified through analysing the ions contributing to the separation of sample groups in the loading scatter plots.

### *In vivo* investigation of GNF-351 inhibition of β-naphthoflavone (BNF)-induced AHR activation

Twenty-one male C57BL/6J mice were divided into three groups: control group (*n* = 7), BNF group (*n* = 7), and BNF + GNF-351 group (*n* = 7). For administration of BNF, mice were given BNF, 5 mg·kg^−1^, dissolved in corn oil. The corresponding vehicle was used as control. For the BNF + GNF-351 group, GNF-351 (5 mg·kg^−1^) dissolved in coin oil was given by oral gavage 5 min before the dose of BNF. The mice were killed 12 h later and the liver, duodenum, jejunum, ileum and colon were taken for mRNA preparation.

### Real-time quantitative PCR measurements of mRNA

Real-time quantitative PCR (qPCR) was performed to investigate the expression of *Cyp1a1* mRNA. RNA was isolated from cell cultures or animal tissues using TRIzol reagent (Invitrogen, Carlsbad, CA, USA), and cDNA generated from 1 μg RNA with a SuperScript II™ Reverse Transcriptase Kit (Life Technologies, Grand Island, NY, USA). qPCR was carried out using SYBR green PCR master mix and ABI Prism 7900HT Sequence Detection System (Applied Biosystems, Foster City, CA, USA).

### Data analysis

The experimental data are given as mean ± SEM. Statistical analysis was carried out using GraphPad Prism 5.0 (GraphPad Software, Inc., La Jolla, CA, USA). Comparisons between two groups were performed using a 2-tailed unpaired Student's *t*-test or Mann–Whitney *U*-tests. Means of more than two groups were compared using one-way anova.

### Materials

Alamethicin, NADPH, UDPGA as uridine 5′-diphosphoglucuronic acid trisodium salt were purchased from Sigma-Aldrich (St. Louis, MO). GNF-351 was synthesized as previously described (Smith *et al*., 2011). All other reagents were of the highest grade commercially available.

## Results

### *In vitro* metabolism profile of GNF-351 in liver and intestine

**Figure 2:**
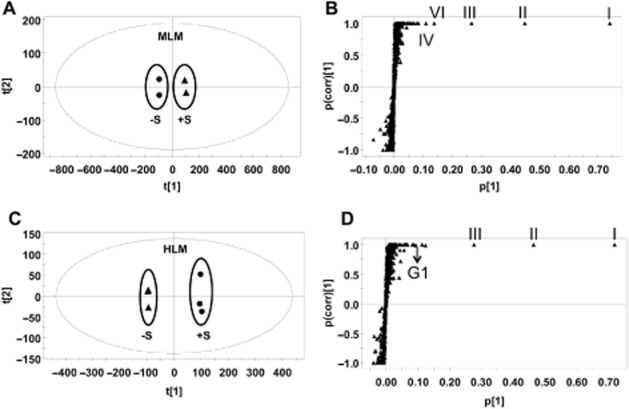
Identification of phase I metabolites in MLMs and phase II metabolites in HLMs. A. Scores plot of a PCA model from MLM phase I incubation mixture without and with GNF-351. B. OPLS loading S-plot of MLM phase I incubation mixture; The p(corr)[1] values represent the interclass difference, and the p[1] values represent the relevant abundance of ions. C. Scores plot of a PCA model from HLM phase II incubation mixture without and with GNF-351. D. OPLS loading S-plot of HLM phase II incubation mixture; The p(corr)[1] values represent the interclass difference, and the p[1] values represent the relevant abundance of ions. Representative ions (I,II, III, IV, VI and G1) were given.

The parent compound, GNF-351, analysed using UPLC-ESI-QTOFMS, eluted at a retention time of 5.5 min (Figure 1B), with an *m/z* of 412.225, in agreement with a match for C_24_H_25_N_7_ with a mass error of 0.0 ppm (Table 1). The main MS/MS fragmentation ions of GNF-351 contained 370, 281, 269, 239, 227 and 144, with the fragmentation pattern shown in Figure 1C. *In vitro* metabolism of GNF-351 was assessed using a microsomal incubation system and the data deconvoluted and analysed by principal components analysis (PCA). This model revealed two clusters corresponding to *in vitro* MLM phase I incubation samples in the presence and absence of GNF-351 (Figure 2A). The OPLS-DA loading plot showed the major ions contributing to the separation containing the parent compounds, the fragments of parent compounds and metabolites (Figure 2B). Detailed information on the I–VIII ions are shown in Table 1, and the trend plot of these ions (I–VIII) displayed in Supporting Information Fig. S1. Separation was also detected between MLM (or HLM) incubation samples and the samples without LMs and without NADPH (Supporting Information Fig. S2). For the glucuronidation incubation system, the PCA model could separate the HLM incubation samples with and without substrates (Figure 2C). Besides the contribution of ions derived from the parent compounds and fragments towards this separation, two glucuronides were also observed to contribute to the separation (Figure 2D and Table 1). The representative chromatograms are displayed in Supporting Information Fig. S3. These two glucuronides were not detected in the MLM incubation system.

**Table 1:** List of detected metabolites in *in vitro* incubation system or mouse faeces

Symbol	RT (min)	Observed m/z	Formula	Mass error (ppm)	Identity
I	5.38	412.225	C_24_H_25_N_7_[H^+^]	0.0	GNF-351
II	5.38	269.154	C_14_H_16_N_6_[H^+^]	9.3	GNF-351 fragment
III	5.38	144.082	C_10_H_9_N[H^+^]	4.9	GNF-351 fragment
IV	5.38	227.105	C_11_H_10_N_6_[H^+^]	2.2	GNF-351 fragment
V	5.38	370.179	C_21_H_19_N_7_[H^+^]	2.7	GNF-351 fragment
VI	5.15	428.219	C_24_H_25_N_7_O[H^+^]	−2.1	Oxidized GNF-351
VII	4.89	428.220	C_24_H_25_N_7_O [H^+^]	−2.1	Oxidized GNF-351
VIII	4.78	370.180	C_21_H_19_N_7_[H^+^]	2.7	Tri-demethylated GNF-351
G1	4.86	588.259	C_30_H_33_N_7_O_6_[H^+^]	3.2	GNF-351 glucuronide
G2	4.12	588.253	C_30_H_33_N_7_O_6_[H^+^]	−7.0	GNF-351 glucuronide
F1	4.60	444.214	C_24_H_25_N_7_O_2_[H^+^]	−1.8	Dioxidized GNF-351
F2	4.19	386.174	C_21_H_19_N_7_O[H^+^]	2.8	Oxidized and tri-demethylated GNF-351
F3	3.75	386.172	C_21_H_19_N_7_O[H^+^]	−2.3	Oxidized and tri-demethylated GNF-351
F4	3.09	402.169	C_21_H_19_N_7_O_2_[H^+^]	3.0	Dioxidized and tri-demethylated GNF-351
F5	4.06	426.204	C_24_H_23_N_7_O[H^+^]	−0.5	Dehydrogenated product of oxidized GNF-351
F6	4.20	524.174	C_24_H_25_N_7_SO_5_[H^+^]	4.6	Sulfation product of dioxidized GNF-351

**Figure 3:**
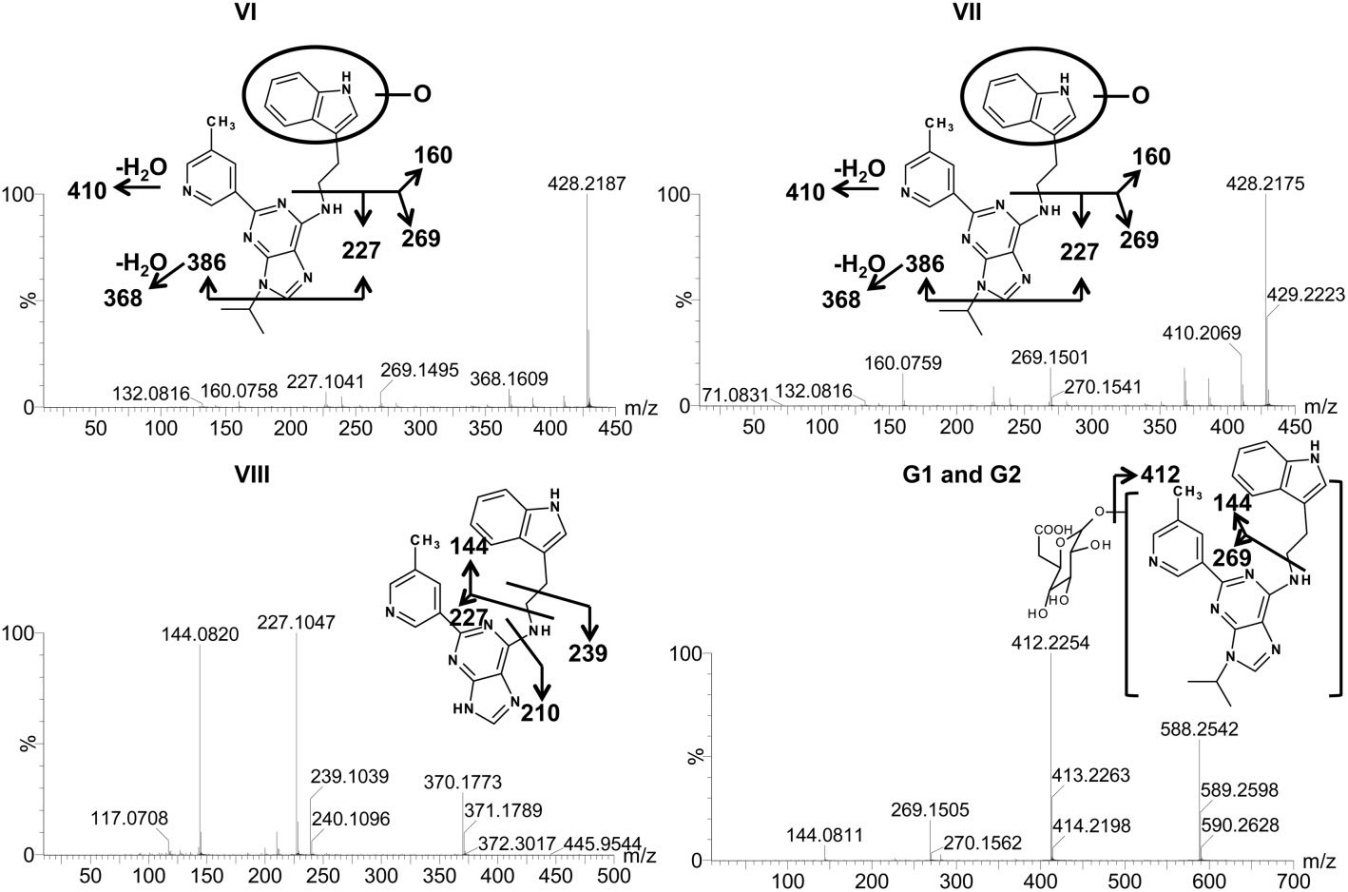
Tandem MS spectrum and proposed chemical structures of representative metabolites VI, VII, VIII and G1(G2). V1, VII, VIII and G1(G2) represent oxidized GNF-351, oxidized GNF-351, tri-demethylated GNF-351, and GNF-351 glucuronide.

The chemical structures of the metabolites were elucidated on the basis of MS/MS fragmentation patterns (Figure 3). The oxidized metabolites VI and VII were identified at retention times of 5.15 and 4.89 min, and gave a match for C_24_H_25_N_7_O with the mass error of −2.1 ppm. The same MS/MS fragments were produced for these two metabolites, containing ions of 410, 386, 368, 269, 227 and 160 Da. Compared with the fragments of the parent compound, the fragment ion 160 was 16 Da higher than one fragment ion of parent compound 144, indicating the addition of an oxygen atom in the upper fraction of GNF-351 as indicated (Figure 3). Ion VIII eluted at 4.78 min, and displayed a protonated molecule at *m/z* 370.180, which gave a match for C_21_H_19_O_7_ with the mass error of 2.7 ppm. The main fragments of metabolite VIII contained 239, 227, 210 and 144 ions. The major fragment of parent compound 269 was not found in the fragments of this metabolite, indicating that metabolite VIII was tri-demethylated GNF-351 and the tri-demethylation reaction occurred in the lower fraction of GNF-351, as shown in Figure 3. The GNF-351 glucuronides (G1 and G2) were eluted at 4.86 and 4.12 min, respectively, and yielded a match for C_30_H_33_N_7_O_6_ with the mass error of 3.2 and −7.0 for G1 and G2. The ion 412 was 176 Da lower than the molecular ion 588, indicating the loss of one molecule of glucuronic acid, further demonstrating the formation of glucuronides. All the detected phase I metabolites were found in the products of the HIM and MIM incubations.

### *In vitro* screening of phase I and phase II enzymes involved in the metabolism of GNF-351

**Figure 4:**
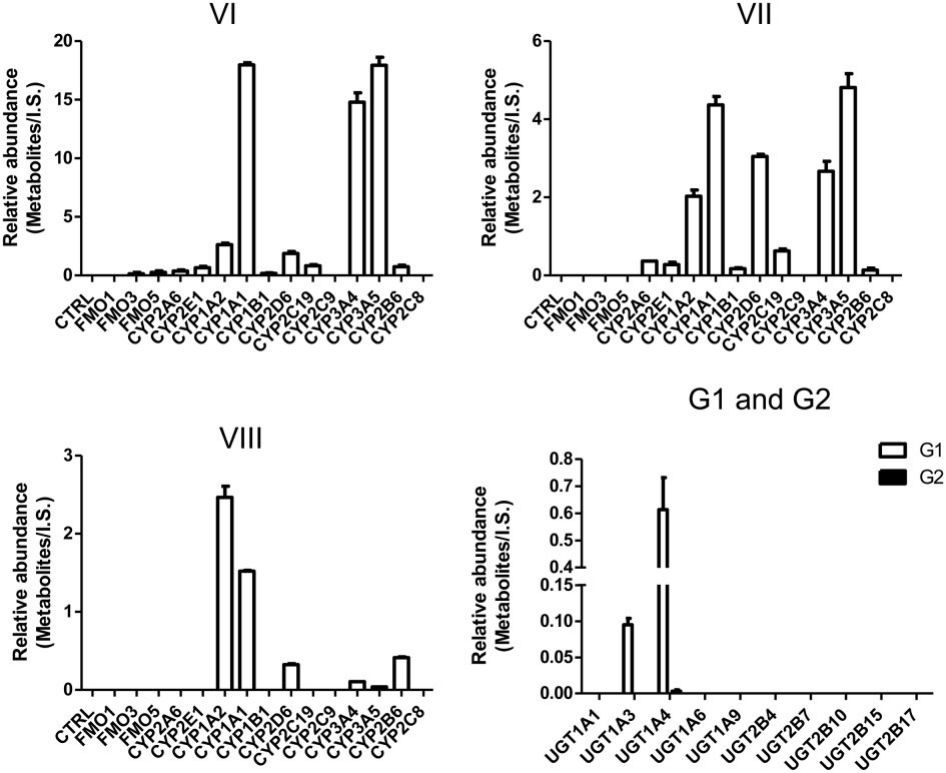
*In vitro* recombinant enzymes screening of phase I DMEs and UGTs involved in the formation of metabolites VI, VII, VIII, G1 and G2. For screening of phase I DMEs, the incubation system contained 50 mM Tris-HCl buffer solution (pH = 7.4), 2 pmol CYP isoforms or 5 mg FMO isoforms, 2 mM MgCl_2_, 100 μM noscapine and 1 mM freshly prepared NADPH. For identification of UGT isoforms, the incubation system (200 μL) contains 50 mM Tris-HCl buffer solution (pH = 7.4), 5 μg of each UGT form, 2 mM MgCl_2_, 100 μM noscapine, 1 mM freshly prepared UDPGA. The relative abundance (peak area ration of metabolites/internal standard) is given.

Among the tested phase I enzymes, CYP1A1/2 and CYP3A4/5 were identified as the major enzymes involved in the formation of metabolite VI (Figure 4). For the formation of metabolite VII, CYP1A1, CYP1A2, CYP2D6, CYP3A4 and CYP3A5 exhibited higher catalytic activities than the other tested phase I enzymes. CYP1A1 and CYP1A2 played a major role in the formation of metabolite VIII. UGT1A4 was demonstrated to be the major UGT form involved in the glucuronidation of GNF-351. UGT1A3 also exerted catalytic activity towards the formation of glucuronide-1.

### Low absorption of GNF-351 and metabolite profiles of GNF-351 in faeces

**Figure 5:**
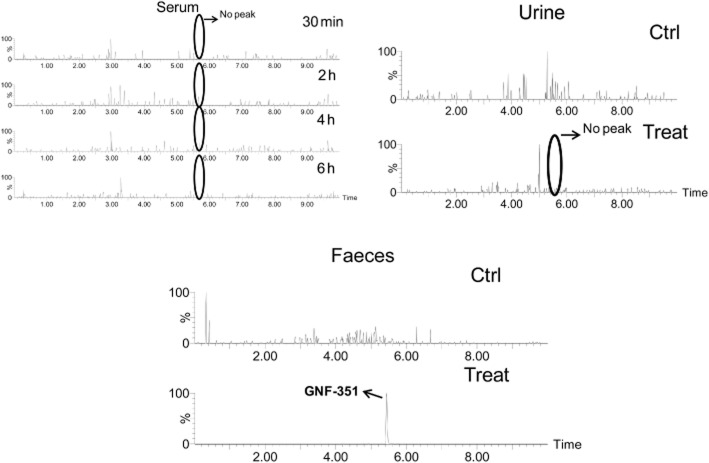
Detection of GNF-351 in serum (0–6 h), urine (24 h) and faeces (24 h). The peak of GNF-351 should appear at a retention time of 5.38 min, and the corresponding molecular ion ( [M + H]^+^ = 412.225) was employed to extract the peak.

**Figure 6:**
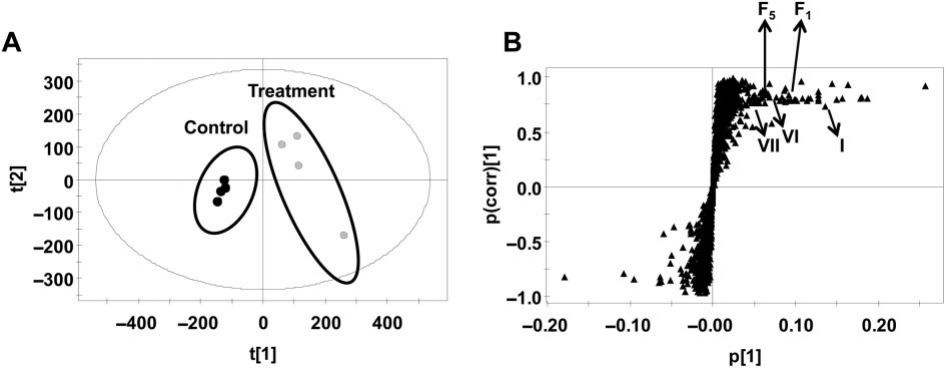
Identification of metabolites in 24 h faeces from mice dosed with 5 mg·kg^−1^ GNF-351. A. Scores plot of a PCA model from mouse faecal samples without and with GNF-351. B. OPLS loading S-plot of mouse faecal samples; The p(corr)[1] values represent the interclass difference, and the p[1] values represent the relevant abundance of ions. Representative ions (I, VI, VII, F1 and F5) were given.

Serum was taken at 0, 0.5, 2.0, 4.0 and 6 h after oral gavage of 5 mg·kg^−1^ GNF-351. GNF-351 was not detected at any of these time points, indicating poor absorption (Figure 5). GNF-351 was also not found in 24 h urine, further indicating poor absorption of GNF-351. Given that GNF-351 can only be detected in faeces (24 h), a metabolomics study was performed to search for metabolites of GNF-351. Separation was observed between the control group and GNF-351-treated group using the PCA model (Figure 6A). From the OPLS-DA loading plot (Figure 6B), new metabolites (F1–F6), in addition to the metabolites detected using the *in vitro* incubation system, were found. The trend plot of F1–F6 is given in Supporting Information Fig. S4.

**Figure 7:**
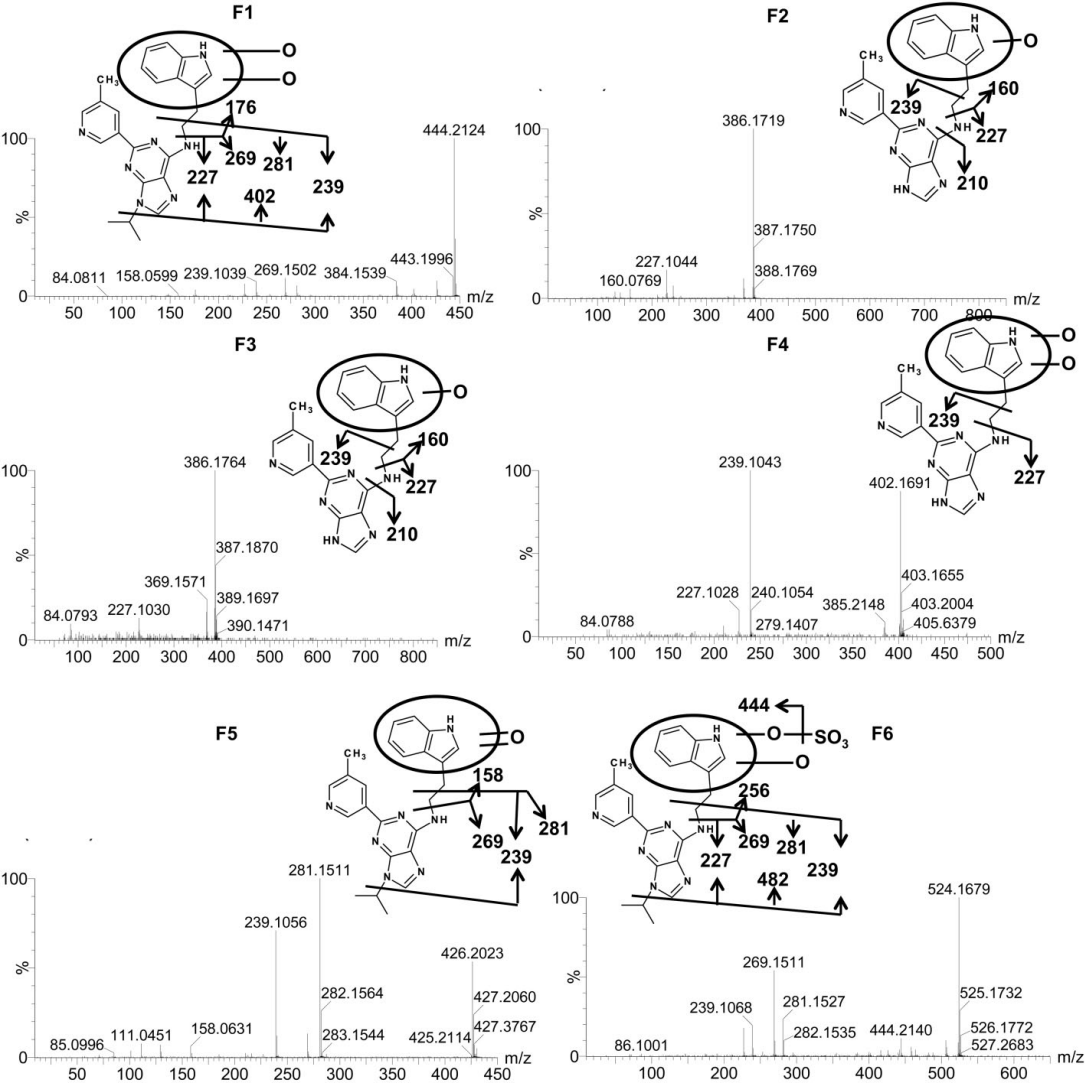
Tandem MS spectrum and proposed chemical structures of metabolites F1 (dioxidized GNF-351), F2 (oxidized and tri-demethylated GNF-351), oxidized and F3 (tri-demethylated GNF-351), F4 (dioxidized and tri-demethylated GNF-351), F5 (dehydrogenated product of oxidized GNF-351), and F6 (sulfation product of dioxidized GNF-351).

F1 eluted at 4.6 min and exhibited a protonated molecule at 444.214 Da. The matched molecular formula was C_24_H_25_N_7_O_2_ (mass error = −1.8 ppm), and the MS/MS fragmentation indicated that this metabolite was di-oxidized GNF-351 in which the di-oxidation reaction occurred in the upper fraction of GNF-351 as indicated (Figure 7). Metabolites F2 (*R*_t_ = 4.19 min, *m/z* = 386.174) and F3 (*R*_t_ = 3.75 min, *m/z* = 386.172) gave a match for C_21_H_19_N_7_O with a mass error of 2.8 and −2.3 ppm respectively. According to the MS/MS fragments and proposed fragmentation mechanism, F2 and F3 were likely to be derived from oxidation and tri-demethylation of GNF-351. Metabolite F4 exhibited a molecular ion at m/z 402.169, with a good match for the molecular formula C_21_H_19_N_7_O_2_. Metabolite F4 was further identified to be di-oxidized and tri-demethylated GNF-351 based on the proposed MS/MS fragmentation mechanism. Metabolite F5 eluted at 4.06 min with the protonated molecule to be 426.204, which was 2 Da less than oxidized GNF-351, thus indicating the formation of a dehydrogenated product of oxidized GNF-351 (Figure 7). The protonated molecule of F6 (*m/z* = 524.174) was 80 Da higher than metabolite F1, indicating sulfation of F1. The fragment of F6 256 was 80 Da higher than the fragment of F1 176, further supporting the occurrence of sulfation in the upper fraction of F1. The proposed structure of F6 is given in Figure 7.

### *In vivo* inhibition by GNF-351 of BNF-induced AHR activation

**Figure 8:**
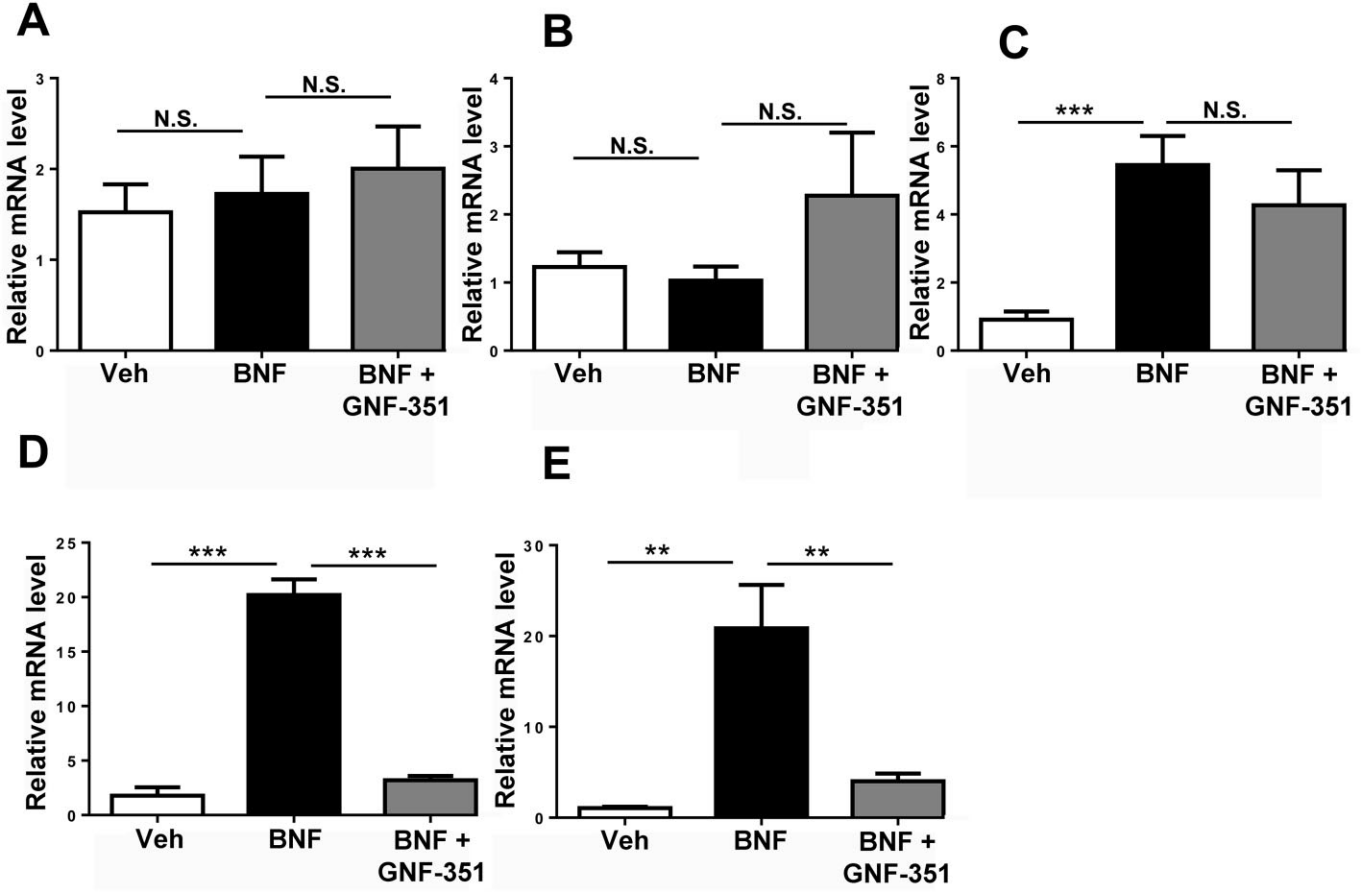
Investigation of the inhibition by GNF-351 of BNF-induced expression of *Cyp1a1* mRNA in duodenum (A), jejunum (B), liver (C), ileum (D), and colon (E). GNF-351 (5 mg·kg^−1^, p.o.) was given 5 min before the administration of BNF (5 mg·kg^−1^, p.o.). The data are means ± SEM (*n* = 7 for each group). **P < 0.01; ***P < 0.001; N.S., not significant. 18S RNA was used as internal standard.

According to an earlier report (Smith *et al*., 2011), GNF-351 exhibited the strongest inhibition potential after a 12 h treatment, and did not show inhibition after 24 h. Thus, the treatment time in our experiments was chosen to be 12 h. BNF (5 mg·kg^−1^) did not induce the expression of *Cyp1a1* mRNA in duodenum (Figure 8A) and jejunum (Figure 8B), but did elevate expression of *Cyp1a1* mRNA in liver (*P* < 0.001, Figure 8C) and ileum (*P* < 0.001, Figure 8D). GNF-351 administration did not inhibit BNF-induced *Cyp1a1* mRNA expression in liver (Figure 8C), but significantly inhibited BNF-induced induction of *Cyp1a1* mRNA in ileum (*P* < 0.001, Figure 8D). A significant induction (*P* < 0.01, Figure 8E) of *Cyp1a1* mRNA expression by BNF was also found in colon after administration of 5 mg·kg^−1^ BNF, and this increase was markedly inhibited by co-administration of GNF-351 (*P* < 0.01, Figure 8E) thus indicating that GNF-351 reached the end of the alimentary tract where it retained its biological activity towards the AHR.

## Discussion

There is a drastic increase in the cost for R&D of a new drug to advance to the clinic, and non-optimal drug ADME (absorption, distribution, metabolism and excretion) properties are important reasons for the high attrition rate of promising candidates. During the early evaluation of drugs, preclinical studies can result in the possible failure of candidates with poor ADME properties (Pellegatti, 2012). In the present study, metabolomics was used to elucidate the metabolic pathway with use of *in vitro* phase I and phase II microsome incubation systems. Several phase I metabolites were identified in human and mouse microsomes, including two oxidized GNF-351 (VI and VII) and one tri-demethylated GNF-351 (VIII). For the glucuronidation of GNF-351, there was a species-related difference between results from human and mouse microsomes. From the MS/MS fragmentation patterns, detailed structural information could not be obtained for the glucuronides (G1 and G2). Given that the structure of GNF-351 contains many nitrogen atoms, the glucuronidation sites might occur in these nitrogen atoms as previously reported (Kaivosaari *et al*., 2011). UGT1A4 was the major UGT form involved in glucuronidation of GNF-351, further supporting the occurrence of the N-glucuronidation reaction. N-glucuronidation of drugs has often been reported to exhibit species difference. Therefore, species scaling from animals to humans for the glucuronidation of GNF-351 should be carefully considered.

Drugs must fulfil some important properties to achieve adequate delivery after oral administration, and intestinal absorption is a key determinant. The present study revealed that GNF-351 was not detected in serum for up to 6 h, indicating that orally administered GNF-351 was not effectively absorbed from the gut either due to poor desolution of the compound, low rates of uptake (transport) and/or high efflux from the gut. Given that the GNF-351 was found in faeces but not in urine at 24 h, the metabolomics analysis was performed on faeces. Besides the phase I metabolites detected in the *in vitro* system, more phase I metabolites were detected, including di-oxidized GNF-351 (F1), oxidized and tri-demethylated GNF-351(F2 and F3), di-oxidized and tri-demethylated GNF-351(F4), and a dehydrogenated product of oxidized GNF-351 (F5). Additionally, a new phase II metabolite, a sulfation product of dioxidized GNF-351 (F6) was detected.

Phase I DMEs involved in the metabolism of GNF-351 were identified to decipher the complete metabolic pathway. Among the tested phase I enzymes, CYP1A1, CYP1A2, CYP3A4 and CYP3A5 were demonstrated to be the main enzymes involved in GNF-351 metabolism. Notably, CYP1A1 and CYP1A2 can be significantly induced via AHR activation (Kohle and Bock, 2007), which accelerates the metabolism of GNF-351, further weakening the AHR antagonist activity of GNF-351. Additionally, CYP3A4 and CYP3A5 are involved in the metabolism of many clinical drugs, and the activity can be easily influenced by compounds, such as the inhibitor of CYP3A4 ketoconazole (Fuchs *et al*., 2013) and the inducers of CYP3A4 and CYP3A5, rifampicin and rifaximin (Ma *et al*., 2007). Therefore, the risk of drug–drug interactions should be considered in the R&D process of GNF-351. Individual differences in these enzymes also could result in different rates of metabolism of GNF-351. For example, women might exhibit higher metabolic activity than men towards GNF-351 because of higher CYP3A4 activity (Scandlyn *et al*., 2008). All these factors would complicate the metabolic response of different individuals towards GNF-351 in different exposure environments. It should be also noted that CYP3A4 is highly expressed in human intestines (Granvil *et al*., 2003), and the present enzyme phenotyping results obtained from recombinant human enzyme sources indicated even more metabolism in human intestines, and thus less antagonistic activity in humans than in mice could be predicted.

As is expected, the *in vivo* efficiency of GNF-351 as an antagonist was limited by poor absorption and complex metabolic factors. Earlier studies have shown that GNF-351 inhibited AHR activation in murine hepatoma-derived reporter cells H1L1.1.1c2 with an IC_50_ value of 116 nM. For a mouse weighing 25 g, its blood volume is 2 mL (8% of body weight). If the oral bioavailability of GNF-351 was 1%, the plasma concentration of GNF-351 was calculated to reach approximately 1.5 μM after oral gavage of 5 mg·kg^−1^ GNF-351. The *in vivo* results indicated that this dose of GNF-351 give orally did not exhibit antagonism of BNF-induced AHR activation in liver, indicating that low absorption and/or rapid metabolism GNF-351 had prevented an adequate concentration of GNF-351 in the liver to bind and inhibit AHR. The antagonism by GNF-351 of intestinal AHR was also investigated. BNF did not significantly activate AHR in the duodenum and jejunum, but exhibited significant activation of AHR in the ileum. While oral GNF-351 did not show measurable inhibition towards BNF-induced AHR activation in liver, it did inhibit BNF-induced AHR activation in ileum and colon.

**Figure 9:**
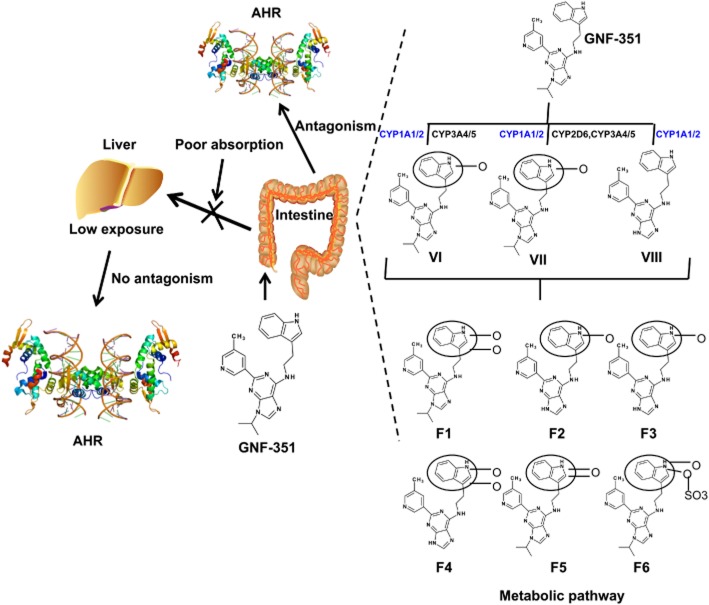
Summary of the present study. Although GNF-351 is an antagonist *in vitro*, its poor absorption and metabolic profile limits its *in vivo* effects. Red colour indicates the CYPs that are significantly affected by AHR activation.

In conclusion, the absorption properties and detailed metabolic pathway of GNF-351 were evaluated in the present study (Figure 9). The results suggested that structural modifications to GNF-351 are required in order to improve its oral absorption and metabolic behaviour. For example, a pro-drug structure, linking GNF-351 to bile acids, might increase oral absorption significantly (Rais *et al*., 2010). Taken together, the information in the present study will be beneficial for further development of GNF-351 as a structure-based antagonist of the AHR in liver.

## Abbreviations

AHR: aryl hydrocarbon receptor
BNF: β-naphthoflavone
CYP: cytochrome P450
DME: drug-metabolizing enzymes
FMO: flavin monooxygenase
HIM: human intestine microsomes
HLM: human liver microsome
MIM: mouse intestine microsomes
MLM: mouse liver microsome
OPLS-DA: orthogonal partial least squares data analysis
PCA: principal components analysis
PCR: real-time quantitative PCR
R&D: research and development
TCDD: 2,3,7,8-tetrachlorodibenzo-*p*-dioxin
UDPGA: uridine 5′-diphosphoglucuronic acid
UGT: UDP-glucuronosyltransferase

## Supporting information

**Figure S1** Trend plot of the ions I-VIII. MLM incubated with (+S) and without (-S) GNF-351. **Figure S2** PCA scores scatter plot for HLMs incubation system (dot), MLMs incubation system (triangle), the incubation without NADPH (diamond), incubation without microsomes (circle), and incubation without GNF-351 (inverted triangle). **Figure S3** Comparison of GNF-351 glucuronidation between HLMs and MLMs. **Figure S4** Trend plot of the ions F1–F6.

## References

[b1] Alexander SPH, Benson HE, Faccenda E, Pawson AJ, Sharman JL, Spedding M (2013). The Concise Guide to PHARMACOLOGY 2013/14: Nuclear Hormone Receptors. Br J Pharmacol.

[b2] Barouki R, Coumoul X, Fernandez-Salguero PM (2007). The aryl hydrocarbon receptor, more than a xenobiotic-interacting protein. FEBS Lett.

[b3] Fang ZZ, Krausz KW, Li F, Cheng J, Tanaka N, Gonzalez FJ (2012). Metabolic map and bioactivation of the anti-tumour drug noscapine. Br J Pharmacol.

[b4] Fernandez-Salguero P, Pineau T, Hilbert DM, McPhail T, Lee SS, Kimura S (1995). Immune system impairment and hepatic fibrosis in mice lacking the dioxin-binding Ah receptor. Science.

[b5] Fuchs I, Hafner-Blumenstiel V, Markert C, Burhenne J, Weiss J, Haefeli WE (2013). Effect of the CYP3A inhibitor ketoconazole on the PXR-mediated induction of CYP3A activity. Eur J Clin Pharmacol.

[b6] Granvil CP, Yu AM, Elizondo G, Akiyama TE, Cheung C, Feigenbaum L (2003). Expression of the human CYP3A4 gene in the small intestine of transgenic mice: *in vitro* metabolism and pharmacokinetics of midazolam. Drug Metab Dispos.

[b7] Johnson CH, Gonzalez FJ (2012). Challenges and opportunities of metabolomics. J Cell Physiol.

[b8] Johnson CH, Patterson AD, Idle JR, Gonzalez FJ (2012). Xenobiotic metabolomics: major impact on the metabolome. Annu Rev Pharmacol Toxicol.

[b9] Kaivosaari S, Finel M, Koskinen M (2011). N-glucuronidation of drugs and other xenobiotics by human and animal UDP-glucuronosyltransferases. Xenobiotica.

[b10] Kilkenny C, Browne W, Cuthill IC, Emerson M, Altman DG (2010). Animal research: reporting *in vivo* experiments: the ARRIVE guidelines. Br J Pharmacol.

[b11] Kim SH, Henry EC, Kim DK, Kim YH, Shin KJ, Han MS (2006). Novel compound 2-methyl-2H-pyrazole-3-carboxylic acid (2-methyl-4-o-tolylazo-phenyl)-amide (CH-223191) prevents 2,3,7,8-TCDD-induced toxicity by antagonizing the aryl hydrocarbon receptor. Mol Pharmacol.

[b12] Kohle C, Bock KW (2007). Coordinate regulation of Phase I and II xenobiotic metabolisms by the Ah receptor and Nrf2. Biochem Pharmacol.

[b13] Li F, Lu J, Cheng J, Wang L, Matsubara T, Csanaky IL (2013). Human PXR modulates hepatotoxicity associated with rifampicin and isoniazid co-therapy. Nat Med.

[b14] Ma X, Shah YM, Guo GL, Wang T, Krausz KW, Idle JR (2007). Rifaximin is a gut-specific human pregnane X receptor activator. J Pharmacol Exp Ther.

[b15] Matsubara T, Tanaka N, Krausz KW, Manna SK, Kang DW, Anderson ER Metabolomics identifies an inflammatory cascade involved in dioxin-and diet-induced steatohepatitis. Cell Metab.

[b16] McGrath J, Drummond G, McLachlan E, Kilkenny C, Wainwright C (2012). Guidelines for reporting experiments involving animals: the ARRIVE guidelines. Br J Pharmacol.

[b17] Pellegatti M (2012). Preclinical *in vivo* ADME studies in drug development: a critical review. Expert Opin Drug Metab Toxicol.

[b18] Rais R, Acharya C, Mackerell AD, Polli JE (2010). Structural determinants for transport across the intestinal bile acid transporter using C-24 bile acid conjugates. Mol Pharm.

[b19] Scandlyn MJ, Stuart EC, Rosengren RJ (2008). Sex-specific differences in CYP450 isoforms in human. Expert Opin Drug Metab Toxicol.

[b20] Smith KJ, Murray IA, Tanos R, Tellew J, Boitano AE, Bisson WH (2011). Identification of a high-affinity ligand that exhibits complete aryl hydrocarbon receptor antagonism. J Pharmacol Exp Ther.

[b21] Veldhoen M, Hirota K, Christensen J, O'Garra A, Stockinger B (2009). Natural agonists for aryl hydrocarbon receptor in culture medium are essential for optimal differentiation of Th17 T cells. J Exp Med.

[b22] Vondracek J, Umannova L, Machala M (2011). Interactions of the aryl hydrocarbon receptor with inflammatory mediators: beyond CYP1A regulation. Curr Drug Metab.

