# Supplementary material for: In vivo effects of the pure aryl hydrocarbon receptor antagonist GNF-351 after oral administration are limited to the gastrointestinal tract
Source: Br J Pharmacol. 2014 Mar 18;171(7):1735–46. doi: 10.1111/bph.12576 (PMC3966752; doi:10.1111/bph.12576)
Supplement: Supplementary file 1 — Figure S1 Trend plot of the ions I-VIII. MLM incubated with (+S) and without (-S) GNF-351. Figure S2 PCA scores scatter plot for HLMs incubation system (dot), MLMs incubation system (triangle), the incubation without NADPH (diamond), incubation without microsomes (circle), and incubation without GNF-351 (inverted triangle). Figure S3 Comparison of GNF-351 glucuronidation between HLMs and MLMs. Figure S4 Trend plot of the ions F1–F6. [file bph0171-1735-sd1.docx]

**SUPPORTING INFORMATION**

**Poor absorption and metabolic properties limit *in vivo* application of the pure aryl hydrocarbon receptor antagonist GNF-351 to the gastrointestinal track**

Zhong-Ze Fang, Kristopher W. Krausz, Kenjiro Nagaoka, Naoki Tanaka, Krishne Gowda, Shantu G. Amin, Gary H. Perdew and Frank J. Gonzalez

**Figure S1.** Trend plot of the ions I-VIII. Mouse liver microsomes (MLM) incubated with (+S) and without (-S) GNF-351.

**Figure S2.** PCA scores scatter plot for human liver microsomes (HLMs) incubation system (dot), mice liver microsomes (MLMs) incubation system (triangle), the incubation without NADPH (diamond), incubation without microsomes (circle), and incubation without GNF-351 (Inverted triangle).

**Figure S3.** Comparison of GNF-351 glucuronidation between human liver microsomes (HLMs) and mice liver microsomes (MLMs).

**Figure S4.** Trend plot of the ions F1-F6.
